# Spontaneous mid‐trimester uterine rupture associated with fetal death in a young patient during COVID‐19 pandemic: A case report

**DOI:** 10.1002/ccr3.6802

**Published:** 2022-12-27

**Authors:** Ahmed Dheyaa Al‐Obaidi, Ahmed Shamil Hashim, Sara Salih, Hashim Talib Hashim

**Affiliations:** ^1^ College of Medicine University of Baghdad Baghdad Iraq

**Keywords:** perinatal mortality, second trimester, spontaneous, uterine rupture

## Abstract

Uterine rupture mostly occurs in the third trimester. However, it may occur at an earlier time with the same catastrophic consequences. The authors present a case report of uterine rupture occurring in the second trimester at 18 weeks gestation.

## Graphical abstract

The most common cause of UR is the existence of an old uterine scar, but in this case, the rapture occurred during the second trimester from a non C/S scar. This is very rare and unusual and it worth urgent operation to save the patient.

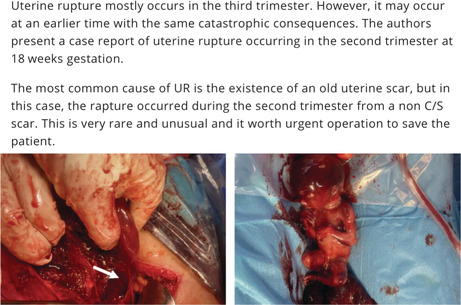

## 1 INTRODUCTION

Uterine rupture (UR) is a rare yet potentially disastrous pregnancy complication.^1^ UR mainly occur in the last trimester of pregnancy, most of which around the time of labor. UR occurring before labor is extremely rare with only a very small percentage occurring in the first or second trimesters.^2^, ^3^ Claeys et al. (2014) have published a meta‐analysis indicating that 80% of UR occur between Weeks 28 and 36 of pregnancy.^4^ UR can be caused by a number of different factors, the most prevalent being a previous cesarean section. Clinical presentation can be misleading as many cases may present with lower or upper abdominal pain or other gastrointestinal and urinary symptoms.^5^ The incidence of UR is 5.1/10,000 delivery in a uterus with previous scarring and 0.7/10,000 delivery in unscarred uteri. UR may lead to catastrophic maternal and neonatal complications; hence, keeping a high index of suspicious is needed and a correlation with imaging modalities findings will greatly aid reaching the diagnosis and implementing the required management.^5^, ^6^ Nevertheless, the present COVID‐19 pandemic poses an additional obstacle to managing patients as every new patient needs COVID‐19 testing and pregnant women requiring immediate delivery without their COVID test undergo delay until their COVID status is confirmed.^7^ Here, we present a case of mid‐trimester UR in a young lady presenting during COVID‐pandemic.

## 2 CASE PRESENTATION

**FIGURE 1:**
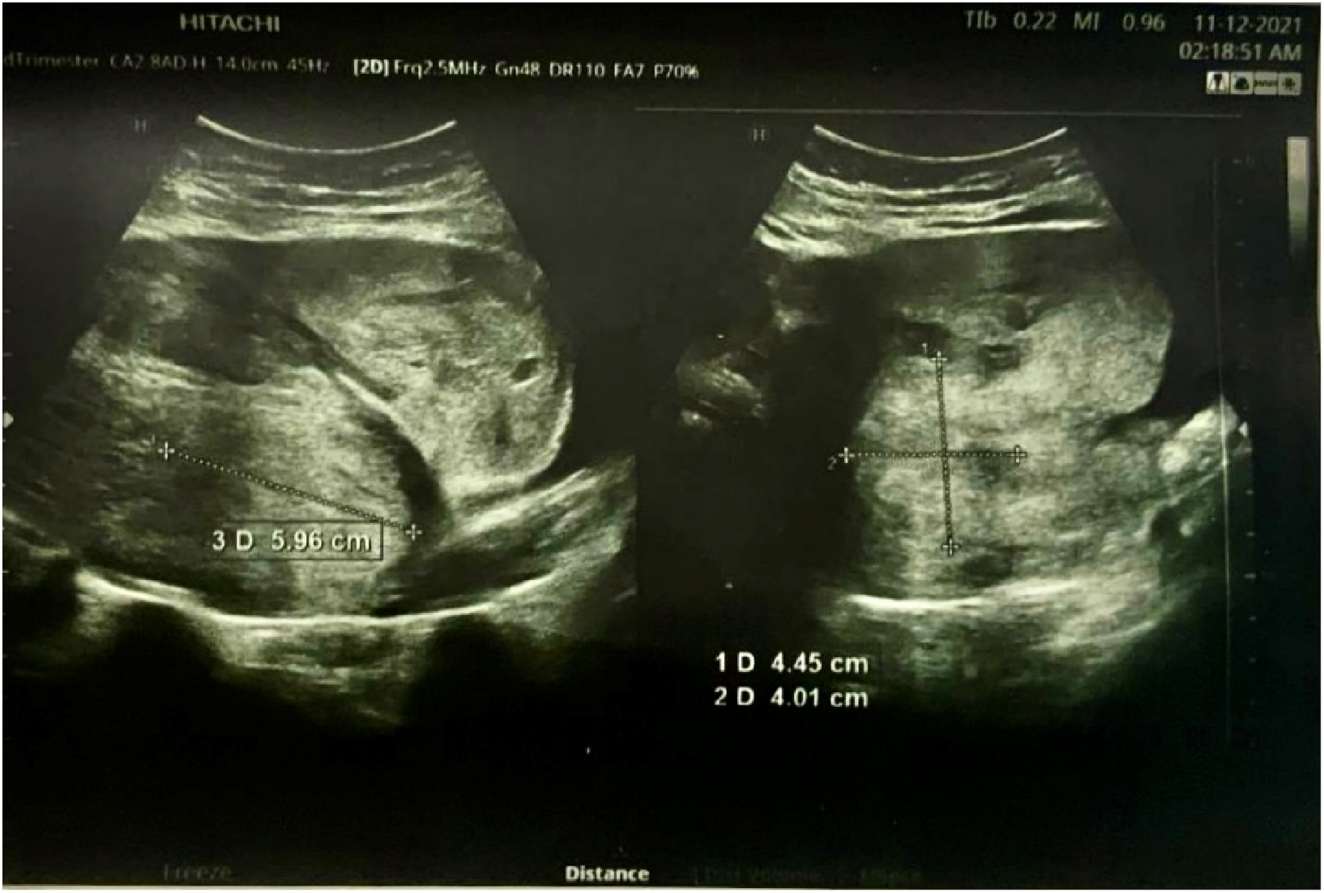
Ultrasound scan of the uterus showing a large blood clot seen at the anterior lower uterine segment between the anterior wall of the uterus and the urinary bladder.

**FIGURE 2:**
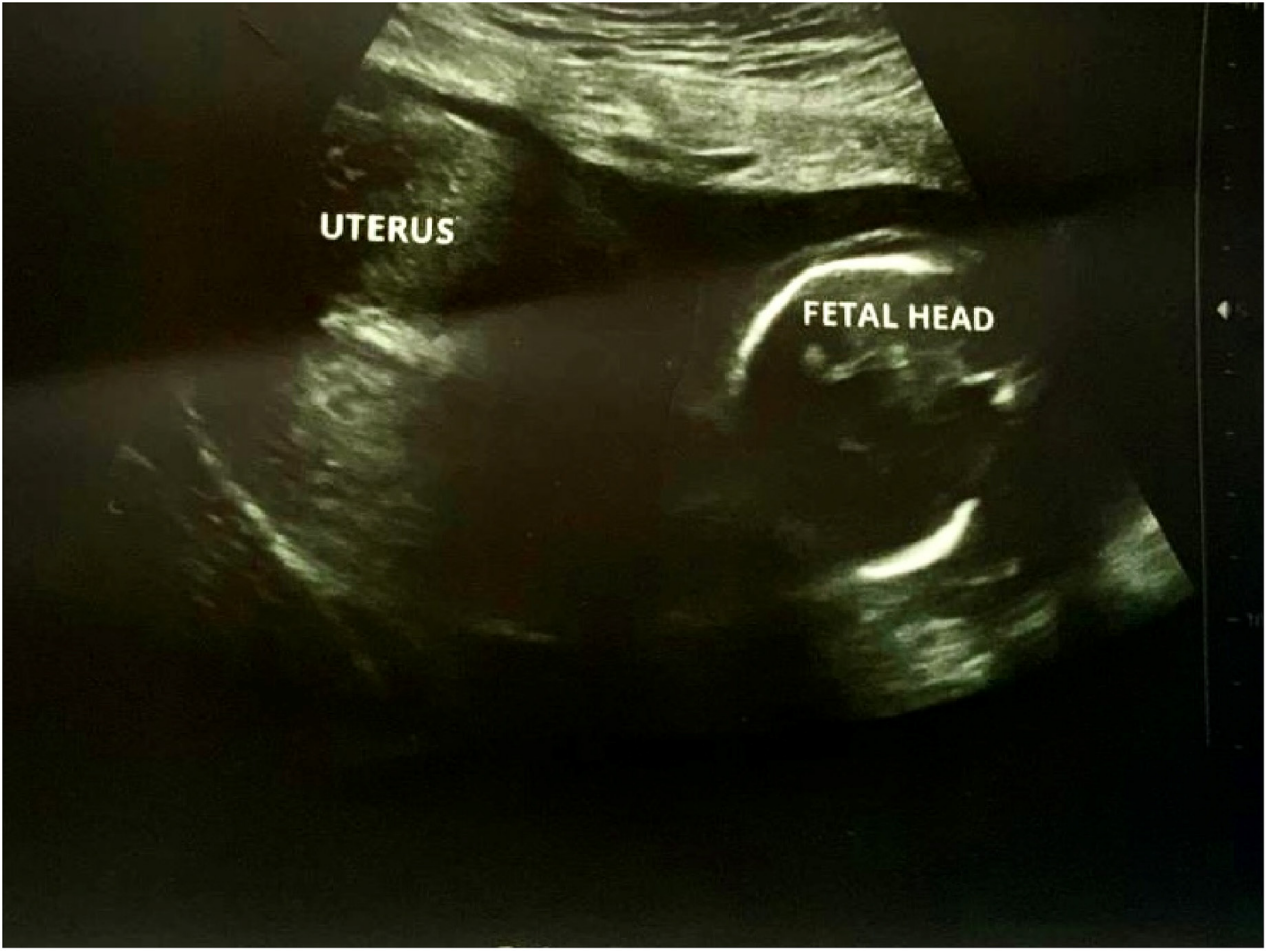
Ultrasound scan of the uterus showing the fetal head outside the uterine cavity.

**FIGURE 3:**
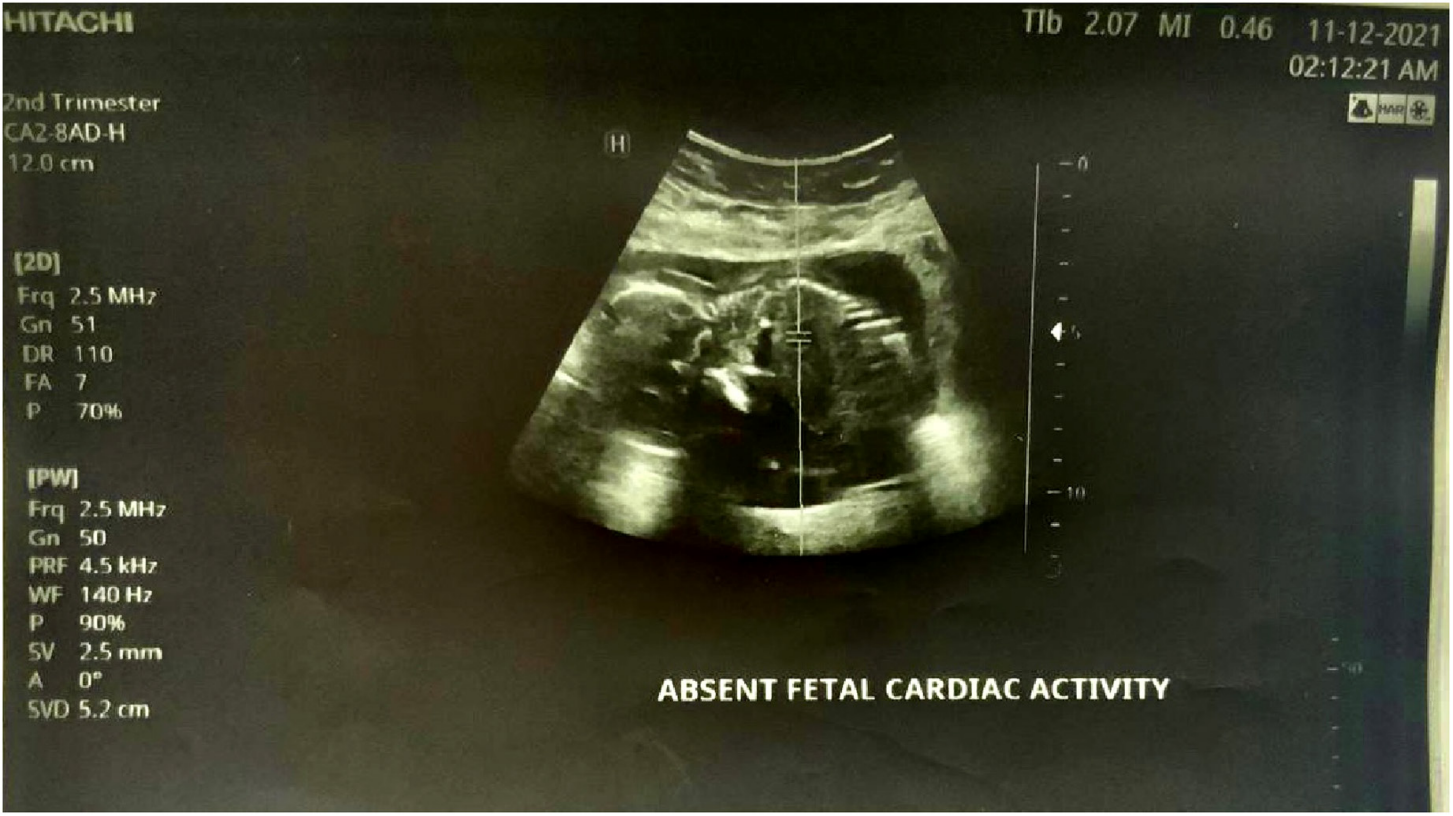
Ultrasound scan showing absent cardiac activity.

We report a case of spontaneous UR occurring at 18 weeks' gestation in a 21‐year‐old woman, who is gravida 3 para 2. She has a history of two prior cesarean sections at term. The first one was performed because of a failure to progress at 38 weeks of gestation and the second one for a “non‐reassuring fetal status” at 39 weeks of gestation. Throughout her current pregnancy, the patient had received routine antenatal care at the hospital, beginning at 10 weeks gestation. Her last visit was the day prior to her presentation and included an ultrasound examination that was normal. Her pregnancy remained uneventful until the patient presented with an abrupt onset of sever persistent lower abdominal pain radiating to the back that was not alleviated by analgesics or rest and was not associated with vaginal bleeding or discharge. Two hours later, she decided to seek consultation and was given empirical treatment for what was suspected to be a urinary tract infection. Her symptoms persisted despite the medications until the following morning, and thus, she went to a hospital where she was referred to the radiology department for an ultrasound. The US revealed a bulky uterus (13.2*7.4 cm) with evidence of full thickness lower anterior uterine wall defect measuring (5 cm) with left lateral extension (4 cm) associated with extrusion of gestational sac containing the fetus into the left iliac fossa. [see Figures 1 and 2] An intracavitary blood clot (6*5*4.5 cm) occupying the lower uterine segment and communicating through the defect with a larger blood clot (9.2*8.5*6.2 cm) were seen anterior to uterus. The fetus was non‐viable on ultrasound with absence of movement and cardiac activity. [see Figure 3] The patient was in severe pain, her temperature was 37.5°C, and she had lower than normal blood pressure (BP 110/60 mmHg) and was tachycardic (HR 110 bpm). Her respiratory rate was 19 breath/cycle, and her hemoglobin was 9 g/dl. Abdominal examination revealed that there was generalized, diffuse tenderness with no bowel sound. Vaginal examination showed that the cervical os was closed and there was slight vaginal bleeding.

Diagnosis was made by correlating clinical findings and imaging techniques as the patient presented with an abrupt onset of sever persistent lower abdominal pain radiating to the back that was not alleviated by analgesics or rest and was not associated with vaginal bleeding or discharge and us showed a large blood clot seen at the anterior lower uterine segment between the anterior wall of the uterus and the urinary bladder so, the diagnosis of a ruptured uterus was made and hence an emergency c/s was necessary.

**FIGURE 4:**
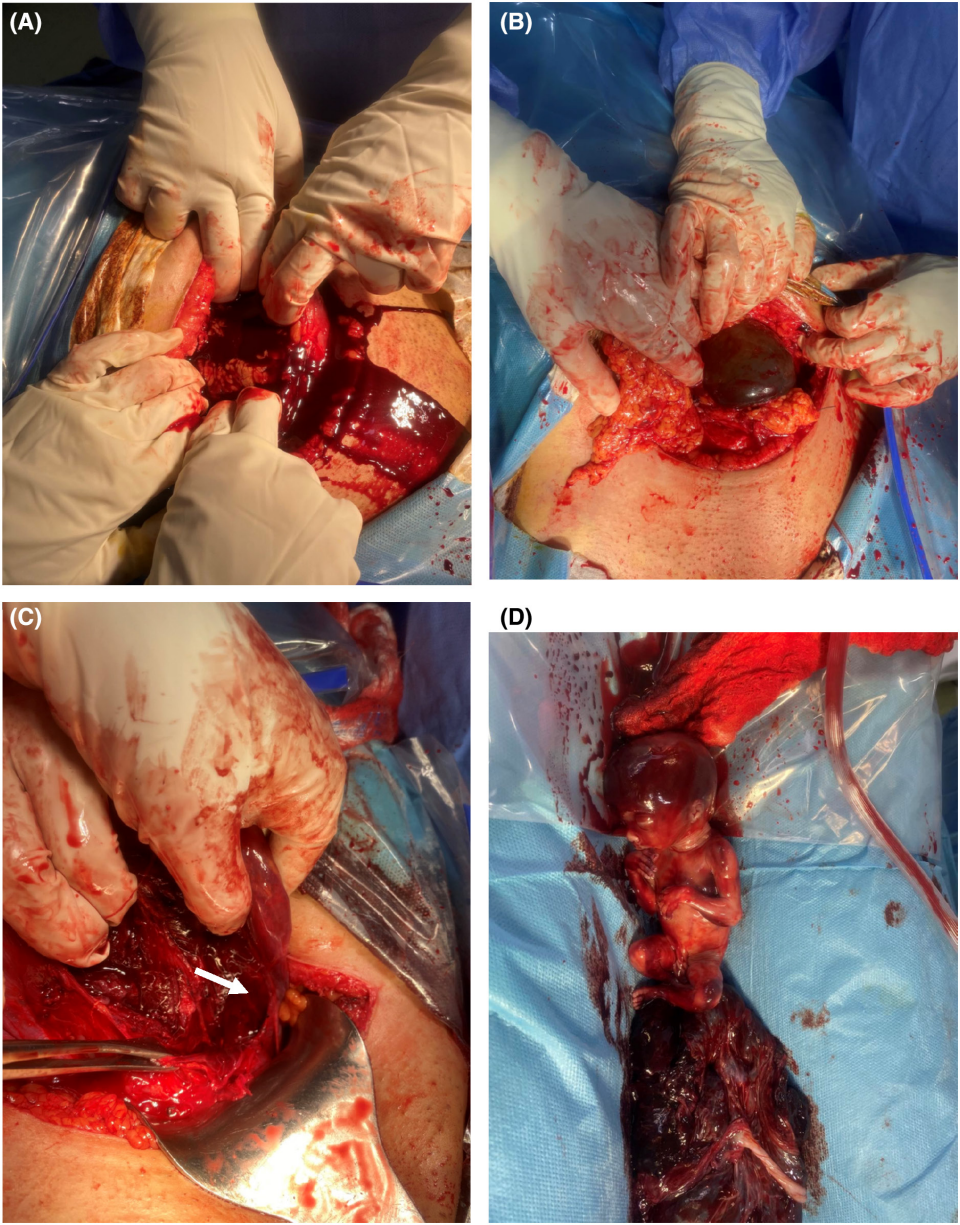
Intra‐operative findings showing: (A) Hemoperitoneum. (B) Uterine rupture at the site of previous incision. (C) inferolateral extension of rupture site (arrow). The forceps is showing the site where the previous scar ends. (D) Dead fetus and the placenta.

The patient, then, was prepared for an emergent laparotomy +/−hysterectomy. Given the current global pandemic of COVID‐19, a quick COVID blood test and throat swab were performed to protect our crew and handle this emergency condition. Both tests verified the patient's infection with COVID. However, she was evidently rushed to the operating room for an emergency laparotomy, while wearing COVID‐19‐specific personal protection equipment. After gaining informed consent for surgical exploration under general anesthesia with possible hysterectomy, a Pfannenstiel‐Kerr incision was made. The intra‐operative findings revealed a ruptured uterus at the same site as one of the previous scars with left lateral extension of 4 cm and moderate hemoperitoneum was encountered. [see Figure 4A–D] The gestational sac harboring the non‐viable fetus was found in the posterior cul‐de‐sac. The ovaries and fallopian tubes appeared healthy and normal on both sides. The bladder was adherent and was dissected off. The rupture site was closed successfully with full thickness double‐layer technique using continuous 1 & 0 polyglactin 910 (Vicryl) sutures. Blood loss was estimated to be 750 ml in total, mostly in clots, which was evacuated. Homeostasis was secured, pelvic wash was done with warm normal saline, and a pelvic drain was inserted in the cul‐de‐sac. The anterior abdominal wall was closed in layers, and the skin was closed in a simple, interrupted manner. Two units of PRBCs (packed red blood cells) and another two units of FFP (fresh frozen plasma) were given to the patient intra‐operatively. Postoperatively, the patient also received one unit of PRBCs and two units of FFP. The patient was discharged from the hospital five days later in good health without experiencing any serious complications. The dead fetus and the placenta were sent for histopathology examination, and the results did not produce any significant findings.

## 3 DISCUSSION

Rupture of the uterus in a non‐laboring woman is highly uncommon and can be fatal for both mother and fetus. Numerous risk factors have been identified in literature including gestational age of more than 40 weeks, advanced maternal age, multiple previous cesarean sections and less than 18 months from the last c‐section. In our 21 years old patient, her only risk factor was the previous cesarean sections.^2^ Additionally, uterine rupture usually occurs in labor, whether spontaneous or induced due to misuse of oxytocin, mismanaged labor, the use of instrumental delivery or obstructed labor^8^; however, it occurred in our patient in her 18th week gestation in a non‐laboring uterus.

Mid‐trimester rupture of the uterus has an incidence of 1 per 5000 deliveries which makes it extremely rare.^1^ The various causes of rupture uterus in second trimester are scarred uterus, grand multipara and an adherent placenta.^4^ Despite the fact that T‐shaped and classical vertical incisions are more prone to subsequent ruptures than the standard modern low transverse approach, and that the fundal area is the most commonly ruptured site in the second trimester‐ low transverse scar rupture usually which occurs in the third trimester,^4^, ^9^ in our patient, the rupture occurred in the second trimester in a transverse lower uterine segment scar which makes it one of the rare cases in literature.

A cohort research examined the role of ultrasonography in the diagnosis of mid‐trimester spontaneous UR. The results postulated that in institutions with timely access to sonography facilities, ultrasound may be used to establish the diagnosis in hemodynamically stable patients and the scan data must be interpreted in the context of clinical findings. The conclusive diagnosis, however, is made during laparotomy, when hemoperitoneum and fetal parts are identified.^5^, ^6^, ^10^ In our case, sonography was able to definitely diagnose the occurrence of uterine rupture and showing he gestational sac outside the uterine cavity which were later confirmed during laparotomy.

The cornerstone of treatment is emergency laparotomy with fetal extraction and hemostasis. Uterine rupture is managed in two ways: either primary repair of the defect or hysterectomy if the bleeding cannot be sufficiently controlled. The clinical scenario, which includes the patient's stability, the location and amount of the defect, as well as the patient's parity and desired future fertility should be considered when deciding the way the patient will be managed. In the majority of cases, total hysterectomy is suggested, especially if the woman has expressed that she has completed her family. On the other side, conservative uterine treatments are more frequently recommended for individuals who are young, have a low parity, and a strong desire to have additional children. Whichever surgical treatment is undertaken, the decision should be made on an individual basis.^5^ In our case, we performed uterine repair as the patient was young with low parity in a clinical setting that made the choice feasible.

## 4 CONCLUSION

The most common cause of UR is the existence of an old uterine scar. Its rarity in the second trimester prior to the onset of labor makes diagnosis extremely difficult. UR must be excluded in all pregnant patients presenting with suspicious symptoms, regardless of trimester they are in to avoid the potentially devastating consequences of a missed diagnosis.

## AUTHOR CONTRIBUTIONS

**Ahmed Dheyaa Al‐Obaidi:** Conceptualization; formal analysis; investigation; resources; validation; writing – original draft. **Ahmed Shamil Hashim:** Conceptualization; data curation; resources; writing – original draft. **Sara Salih:** Conceptualization; data curation; project administration; resources; writing – original draft; writing – review and editing. **Hashim Talib Hashim:** Data curation; project administration; supervision; writing – review and editing.

## FUNDING INFORMATION

No funding was received from any organization.

## CONFLICT OF INTEREST

The author reports no conflicts of interest in this work.

## CONSENT

Written informed consent was obtained from the patient's mother to publish this report in accordance with the journal's patient consent policy.

## Data Availability

Data of this case report will be available on a reasonable request from the corresponding author.
